# Modulation of *Caenorhabditis elegans* infection sensitivity by the LIN-7 cell junction protein

**DOI:** 10.1111/j.1462-5822.2012.01824.x

**Published:** 2012-06-21

**Authors:** XiaoHui Sem, Jason F Kreisberg, Trupti Kawli, Man-Wah Tan, Mikael Rhen, Patrick Tan

**Affiliations:** 1 Genome Institute of SingaporeSingapore, 138672, Singapore; 2 Department of Microbiology, Tumor and Cell Biology, Karolinska InstitutetStockholm, 17177, Sweden; 3 Department of Genetics, Stanford University School of MedicineStanford, CA, 94305, USA; 4 Department of Microbiology and Immunology, Stanford University School of MedicineStanford, CA, 94305, USA; 5 Department of Microbial Pathogenesis, Genentech, Inc.South San Francisco, CA, 94099, USA; 6 Duke-NUS Graduate Medical School SingaporeSingapore, 169547, Singapore; 7 Cancer Sciences Institute of Singapore, National University of SingaporeSingapore, 117456, Singapore

## Abstract

In *Caenorhabditis elegans*, the LIN-2/7/10 protein complex regulates the activity of signalling proteins. We found that inhibiting *lin-7* function, and also *lin-2* and *lin-10*, resulted in enhanced *C. elegans* survival after infection by *Burkholderia* spp., implicating a novel role for these genes in modulating infection outcomes. Genetic experiments suggested that this infection phenotype is likely caused by modulation of the DAF-2 insulin/IGF-1 signalling pathway. Supporting these observations, yeast two-hybrid assays confirmed that the LIN-2 PDZ domain can physically bind to the DAF-2 C-terminus. Loss of *lin-7* activity also altered DAF-16 nuclear localization kinetics, indicating an additional contribution by *hsf-1*. Unexpectedly, silencing *lin-7* in the hypodermis, but not the intestine, was protective against infection, implicating the hypodermis as a key tissue in this phenomenon. Finally, consistent with *lin-7* acting as a general host infection factor, *lin-7* mutants also exhibited enhanced survival upon infectionby two other Gram-negative pathogens, *Pseudomonas* and *Salmonella* spp.

## Introduction

The soil nematode *Caenorhabditis elegans* has been used as a model system to dissect host–pathogen interactions for many microbes, including *Burkholderia, Pseudomonas* and *Salmonella* spp. (Aballay *et al*., 2000; Labrousse *et al*., 2000; Gan *et al*., 2002). In many cases, bacterial mutants exhibiting reduced virulence in mice also displayed attenuated virulence in the nematode, supporting the suitability of *C. elegans* as a model for studying selected aspects of mammalian host–pathogen interactions (Tan *et al*., 1999; Gan *et al*., 2002; Tenor *et al*., 2004). Use of the *C. elegans* model has also delineated several conserved host pathways modulating infection outcomes, including the p38 mitogen-activated protein kinase (MAPK), TGF-β and DAF-2 insulin/IGF-1 signalling pathways (Kim *et al*., 2002; Mallo *et al*., 2002; Garsin *et al*., 2003).

Upon pathogen exposure, activation of host defence pathways occur in a co-ordinated and integrated manner to elicit effective and appropriate host responses (Alper *et al*., 2007). Previous research has shown that many components of these host defence pathways are broadly expressed, making it likely that they need to be kept under strict regulatory control across different cells and tissues. Reflecting the importance of tissue specificity, different cellular compartments in *C. elegans* have also been shown to exhibit distinct pathogen responses under specific challenges. For example, in the intestine, p38 MAPK regulates the *lys-2* lysozyme during bacterial infection (Ren *et al*., 2009); while in the hypodermis, p38 MAPK regulates the antimicrobial peptide *nlp-29* during antifungal responses (Pujol *et al*., 2008; Ziegler *et al*., 2009).

DAF-2 insulin/IGF-1 signalling is one of the most extensively studied pathways regulating host infection outcomes in *C. elegans*. DAF-2 signalling is also involved in modulating lifespan, dauer formation and resistance to abiotic stresses (Kenyon *et al*., 1993; Murakami and Johnson, 1996; Kimura *et al*., 1997; Barsyte *et al*., 2001). Activation of the DAF-2 receptor tyrosine kinase (RTK) initiates a downstream phosphatidylinositol 3-kinase (PI3K) pathway, involving the AKT-1 kinase (Paradis and Ruvkun, 1998), which culminates in the phosphorylation of DAF-16, a forkhead-related FOXO transcription factor, and subsequent egression of phosphorylated DAF-16 from the nucleus into the cytoplasm (Lin *et al*., 1997; Ogg *et al*., 1997). DAF-16 regulates genes involved in stress responses, innate immunity and antimicrobial function (Murphy *et al*., 2003) and mediates the phenotypes exhibited by *daf-2* loss-of-function mutants (Kenyon *et al*., 1993; Garsin *et al*., 2003). Besides DAF-16, the DAF-2 signalling pathway also affects heat shock factor 1 (HSF-1), a transcription factor regulating several heat shock proteins (HSPs) involved in the heat shock response. Upon induction, HSPs act as chaperones binding to unfolded or damaged proteins (Frydman, 2001). Similar to DAF-16, HSF-1 also contributes to the longevity of *daf-2* mutants and their enhanced ability to survive pathogenic assaults (Hsu *et al*., 2003; Singh and Aballay, 2006).

In this study, we performed a targeted reverse genetic screen to identify new genes involved in modulating *C. elegans* infection outcomes. Among the genes screened, we identified *lin-7*, a cell junction gene (Simske *et al*., 1996), as a factor important for influencing *C. elegans* survival upon infection with bacterial pathogens. Genetic and biochemical experiments revealed that *lin-7* modulates the DAF-2 insulin/IGF-1 signalling pathway, by binding directly to the DAF-2 RTK via LIN-2, and that both *daf-16* and *hsf-1* are required for the enhanced survival exhibited by *lin-7* mutants upon infection. We also found that *lin-7* functions predominantly in the *C. elegans* hypodermis to modulate infection outcomes. Our results thus reveal a regulatory connection between LIN-7 and the DAF-2 signalling pathway, and suggest an important role for the hypodermis in dictating the survival outcome of a nematode during bacterial infection.

## Results

### *Burkholderia thailandensis* accumulates in the *C. elegans* intestine during infection

**Fig. 1:**
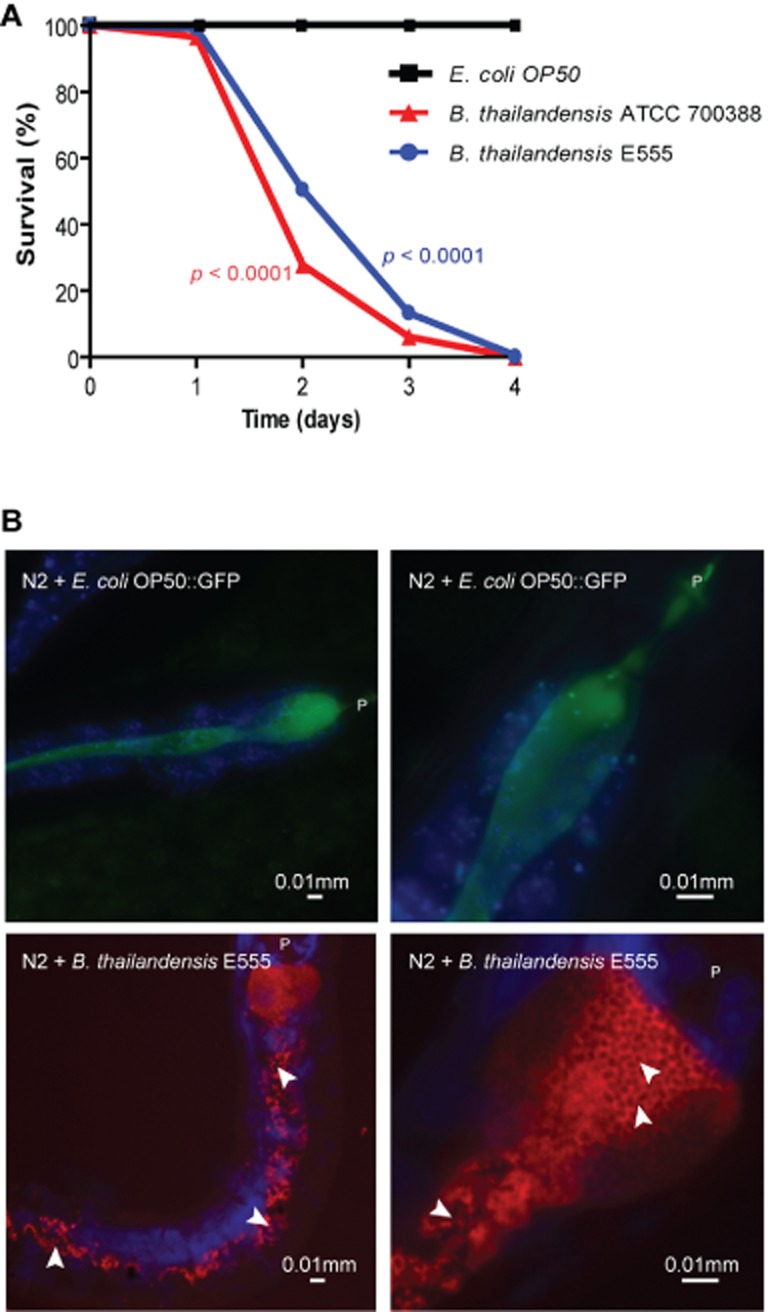
During infection, *B. thailandensis* accumulates in the *C. elegans* intestine. A. Survival of wild-type nematodes was compared when fed *E. coli* OP50, *B. thailandensis* ATCC 700388 (*P* < 0.0001) or *B. thailandensis* E555 (*P* < 0.0001; all *P* values as compared with *E. coli* OP50). Each survival curve is representative of three independent experiments, each with three plates per strain and 40 nematodes per plate. B. Wild-type nematodes were fed *E. coli* OP50::GFP for 8 h (top). Live nematodes were subsequently mounted for microscopy in PBS with NaN_3_. In these merged images, *E. coli* OP50 is shown in green and intestinal autofluorescence in blue. Wild-type nematodes were exposed to *B. thailandensis* E555 for 8 h (bottom). Infected nematodes were then fixed, permeabilized and labelled with monoclonal antibody 3015 and DAPI. In these merged images, *B. thailandensis* E555 is shown in red and DAPI in blue. Images are shown at 40× (left) or 100× magnification (right). The pharynx (P) and individual bacteria (white arrowheads) are indicated. Scale bar represents 0.01 mm.

Although *Burkholderia* spp. have been shown to kill *C. elegans* (O'Quinn *et al*., 2001; Gan *et al*., 2002; Köthe *et al*., 2003), the specific routes of infection used by *Burkholderia* spp. to infect nematodes have not been clearly determined. To address this, we infected nematodes with *Burkholderia thailandensis* E555, a strain expressing a capsular polysaccharide detectable by the monoclonal antibody 3015. Staining infected nematodes with the antibody 3015 allows visualization of each individual bacterium, defined by a clear circular band (Sim *et al*., 2010). Wild-type N2 nematodes infected with *B. thailandensis* E555, or with the reference strain *B. thailandensis* ATCC 700388, exhibited rapid death, with 100% of the nematodes demonstrating lethality after 4 days (*P* < 0.0001, Fig. 1A). Control nematodes fed with GFP-expressing *Escherichia coli* OP50 exhibited a broadly diffuse green fluorescence throughout the intestinal lumen, reflecting efficient bacterial destruction by the pharyngeal grinder (Fig. 1B, top panel). In contrast, immunofluorescence assays of *B. thailandensis* E555-infected nematodes revealed clearly defined intact *B. thailandensis* bacteria throughout the intestinal lumen from 8 h (Fig. 1B, bottom panel) until 32 h post infection (Fig. S1).

### A reverse genetic screen identifies *lin-7* as a host factor to *B. thailandensis* infection

Since *B. thailandensis* was detected in the nematode intestinal lumen, the intestine may represent an important site for host–pathogen interactions. We hypothesized that screening genes expressed in the intestine might identify factors important in modulating host infection outcomes. Querying a publicly accessible database (WormBase, release WS180), we selected 81 genes reported to be expressed in nematode intestinal cells (Table S2). Using RNA interference (RNAi) (Timmons and Fire, 1998), we systematically tested each of the 81 genes for its effect in modulating nematode survival during infection. Briefly, wild-type nematodes were treated with *E. coli* HT115 clones producing gene-targeting double-stranded RNA (dsRNA) (Fire *et al*., 1998), followed by exposure to *B. thailandensis* (see *Experimental procedures*). We specifically identified genes that, when silenced, resulted in prolonged *C. elegans* survival during infection. This strategy eliminates potential confounding factors due to general sickness caused by specific RNAi treatment, which might result in nematodes exhibiting decreased survival during infection.

**Fig. 2:**
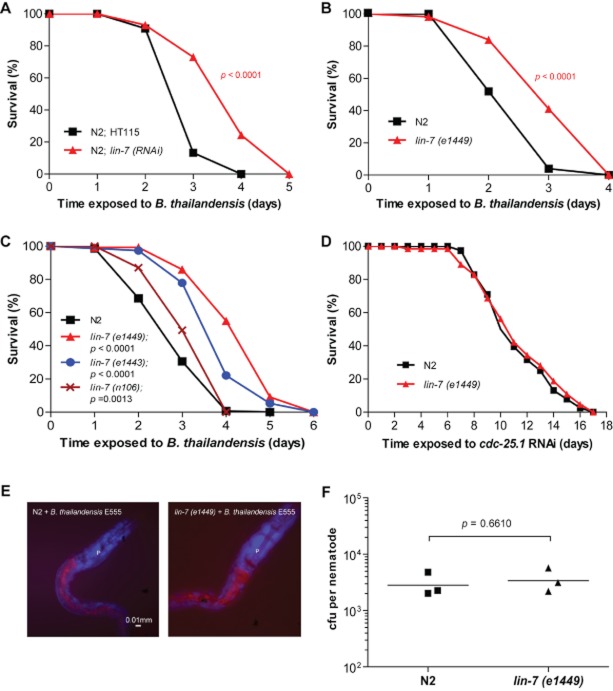
Loss of *lin-7* confers enhanced survival upon *B. thailandensis* infection. A. Wild-type nematodes were first grown on parental *E. coli* HT115 or exposed to *lin-7* dsRNA before transferring to plates containing *B. thailandensis* ATCC 700388 (*P* < 0.0001). B. Wild-type or *lin-7 (e1449)* nematodes were exposed to *B. thailandensis* ATCC 700388 (*P* < 0.0001). C. Wild-type, *lin-7 (e1449)* (*P* < 0.0001), *lin-7 (e1413)* (*P* < 0.0001) or *lin-7 (n106)* (*P* = 0.0013; all *P* values as compared with wild-type) nematodes were first exposed to *cdc-25.1* dsRNA before transferring to plates containing *B. thailandensis* ATCC 700388*.* D. Wild-type or *lin-7 (e1449)* nematodes were first exposed to *cdc-25.1* dsRNA until day 1 adult stage and subsequently after every 2 days, transferred to fresh plates containing the same HT115 strain for lifespan studies (*P* = 0.5661). E. Wild-type or *lin-7 (e1449)* nematodes were exposed to *B. thailandensis* E555 for 24 h. Infected nematodes were then fixed, permeabilized and labelled with monoclonal antibody 3015 and DAPI. In these merged images, *B. thailandensis* E555 is shown in red and DAPI in blue. Images are shown at 40× magnification (right) and the pharynx (P) is indicated. Scale bar represents 0.01 mm. F. Wild-type (▪) or *lin-7 (e1449)* (▴) nematodes were exposed to *B. thailandensis* ATCC 700388 for 24 h (*P* = 0.6610). Infected nematodes were then lysed mechanically and chemically to release intestinal bacteria. Lysates were plated on LB agar with gentamicin and amount of live bacteria per nematode was determined by cfu counts. Each symbol represents the average of 25–35 nematodes and horizontal lines indicate the geometric mean of triplicates.

Of the 81 genes screened, RNAi knock-down of 4 genes (*lin-7, myo-3, rfp-1* and *rrt-2*) conferred significant enhancements of survival (data not shown). Among these, we selected *lin-7*, encoding a cell junction protein (Simske *et al*., 1996), for further analysis. Wild-type nematodes treated with *lin-7* dsRNA survived significantly longer than nematodes treated with parental *E. coli* HT115 upon *B. thailandensis* infection (*P* < 0.0001, Fig. 2A). To verify the use of parental *E. coli* HT115 as a control, wild-type nematodes treated with either parental *E. coli* HT115 or HT115 with an empty RNAi vector did not differ in their susceptibilities to *B. thailandensis* infection (*P* = 0.2896, Fig. S2).

To validate the RNAi results, we repeated the infection assays using the *lin-7 (e1449)* loss-of-function mutant (Ferguson and Horvitz, 1985). The *lin-7* genetic mutant also survived significantly longer than the wild-type nematode upon exposure to *B. thailandensis* (*P* < 0.0001, Fig. 2B).

Previous studies have focused on LIN-*7*'s role in *C. elegans* vulval epithelial cells, where it modulates the localization and activity of LET-23 RTK, a regulator of vulval development (Simske *et al*., 1996; Kaech *et al*., 1998). *lin-7 (e1449)* mutants are vulvaless and produce a characteristic ‘bag of worms’ phenotype (Ferguson and Horvitz, 1985). To address whether the *lin-7*-mediated infection phenotype was confounded by *lin-7* mutants being prone to matricidal hatching, we treated an allelic series of three independent *lin-7* loss-of-function genetic mutants, *lin-7 (e1449), lin-7 (e1443)* and *lin-7 (n106)* (Ferguson and Horvitz, 1985), with *cdc-25.1* dsRNA to render them sterile (Evans *et al*., 2008; Shapira and Tan, 2008). Similar to our observations for fertile nematodes, sterile *lin-7 (e1449)* (*P* < 0.0001), *lin-7 (e1413)* (*P* < 0.0001) and *lin-7 (n106)* (*P* = 0.0013, Fig. 2C) mutants also exhibited enhanced survival during infection compared with sterile wild-type nematodes.

Previous research has shown that *C. elegans* pathways regulating lifespan are also involved in the modulation of host infection outcomes (Garsin *et al*., 2003), leading to speculations that both biological processes are controlled by the same underlying genetic mechanisms (Lithgow, 2003). However, subsequent work reported that enhanced survival during infection is not just a given consequence of longevity (Evans *et al*., 2008). We confirmed that the *lin-7-*mediated infection phenotype is not likely a secondary consequence of lifespan extension because the lifespan of sterile *lin-7 (e1449)* mutants was comparable to that of sterile wild-type nematodes grown on non-pathogenic *E. coli* (*P* = 0.5661, Fig. 2D). These results suggest a hitherto undescribed role for *lin-7* during bacterial infection.

To further define the infection phenotype exhibited by *lin-7* mutants, we performed immunofluorescence assays as described above. At 24 h post infection, we could not observe any significant difference in the intestinal colonization profiles between infected wild-type nematodes and *lin-7 (e1449)* mutants (Fig. 2E). Similar results were obtained when we directly measured intestinal *B. thailandensis* loads by quantifying colony-forming units (cfu) from infected nematodes – there was no significant difference between the abilities of the wild-type nematode and the *lin-7* mutant to limit intestinal pathogen growth (*P* = 0.6610, Fig. 2F). The observation that the pathogen load was not substantially different between the wild-type nematode and the *lin-7* mutant suggests that the enhanced survival exhibited by the *lin-7* mutant is unlikely due to a more efficient restriction on intestinal bacterial growth.

### LIN-7-associated LIN-2 binds to DAF-2 RTK

**Fig. 3:**
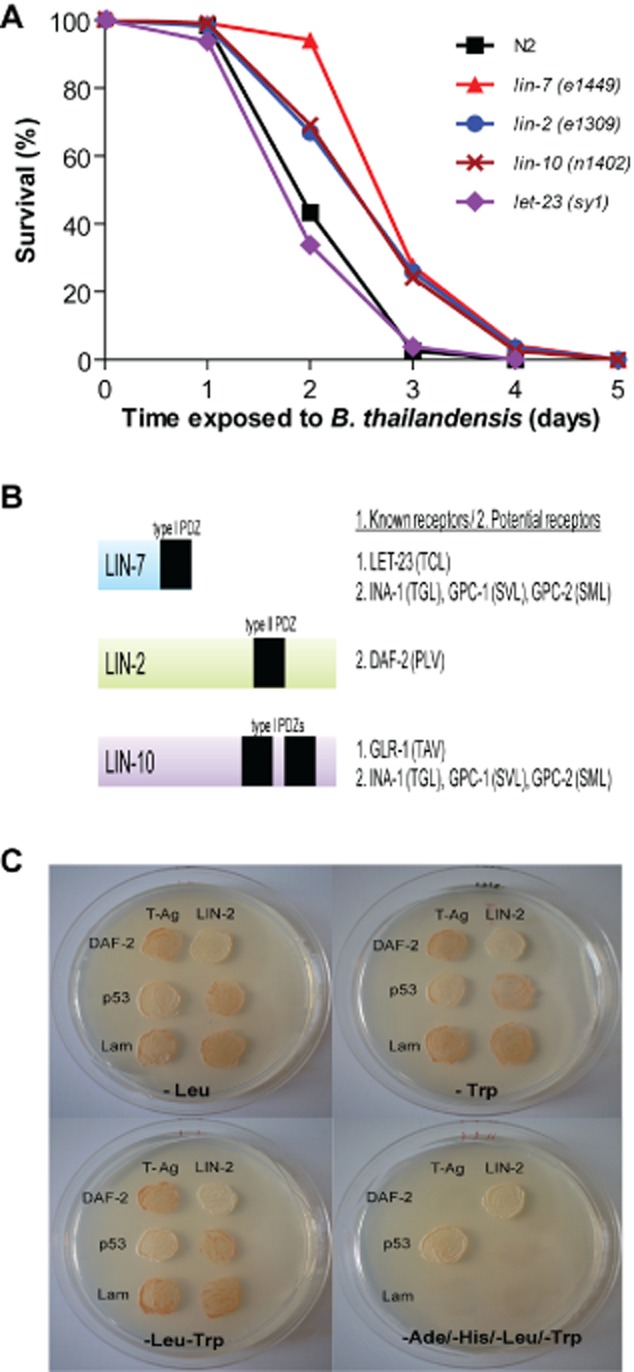
*lin-7, lin-2* and *lin-10* mutants exhibit similar infection phenotypes. A. Wild-type, *lin-7 (e1449)* (*P* < 0.0001), *lin-2 (e1309)* (*P* < 0.0001), *lin-10 (n1402)* (*P* < 0.0001) or *let-23 (sy1)* (*P* = 0.1864; all *P* values as compared with wild-type) nematodes were exposed to *B. thailandensis* ATCC 700388. B. Diagram highlighting the PDZ domains (shaded in black) of LIN-7, LIN-2 and LIN-10 proteins along with (1) known or (2) potential interacting receptors. The PDZ binding consensus motifs at the C-terminals of interacting partners are indicated in parentheses. C. The PDZ domain of LIN-2 (amino acids 288–647) and the C-terminus of DAF-2 (amino acids 1445–1843) were expressed as fusion proteins with the GAL4 AD and DNA-BD respectively. SV40 large T antigen (T-Ag) and murine p53 (p53), as fusion proteins with the same GAL4 AD and DNA-BD, respectively, served as positive controls; human lamin C (Lam), fused to the GAL4 DNA-BD, was used as a negative control. Individually transformed haploid yeast cells were mated and diploids with positive protein–protein interactions were selected on synthetically defined media lacking leucine (-Leu), tryptophan (-Trp), adenine (-Ade) and histidine (-His).

LIN-7 physically associates with two other proteins, LIN-2 and LIN-10, to form a multi-protein complex regulating the function of signalling receptors (Kaech *et al*., 1998; Kim and Sheng, 2004; Alewine *et al*., 2007). To determine if the LIN-2/7/10 complex modulates infection outcomes, we subjected *lin-2* and *lin-10* loss-of-function mutants to *B. thailandensis* infection. Similar to *lin-7* mutants*, lin-2 (e1309)* (*P* < 0.0001) and *lin-10 (n1402)* (*P* < 0.0001) mutants (Horvitz and Sulston, 1980; Ferguson and Horvitz, 1985; Hoskins *et al*., 1996) also survived significantly longer than wild-type nematodes upon *B. thailandensis* infection (Fig. 3A ). These results suggest that LIN-7, LIN-2 and LIN-10 may function together as a tripartite complex to modulate infection outcomes.

In the *C. elegans* vulva, the LIN-2/7/10 complex has been shown to regulate the localization and activity of the LET-23 RTK in epithelial cells (Kaech *et al*., 1998). However, a *let-23 (sy1)* loss-of-function mutant did not exhibit enhanced survival when compared with wild-type nematodes upon *B. thailandensis* infection (*P* = 0.1864, Fig. 3A). This result indicates that the LET-23 RTK is unlikely to mediate the *lin-7*-associated infection phenotype during *B. thailandensis* infection.

LIN-7, LIN-2 and LIN-10 contain postsynaptic density-95, disc large, zona occludens (PDZ) domains that can interact with the C-terminals of receptors (Kaech *et al*., 1998). LIN-7 and LIN-10 contain type I PDZ domains that bind consensus motifs (S/T)*X*(V/I/L), while LIN-2 has a type II PDZ domain which binds Φ-*X*-Φ motifs, where Φ is a hydrophobic residue (Songyang *et al*., 1997). We therefore hypothesized that during infection, the LIN-2/7/10 complex might interact with other signalling receptors to regulate their function.

**Fig. 4:**
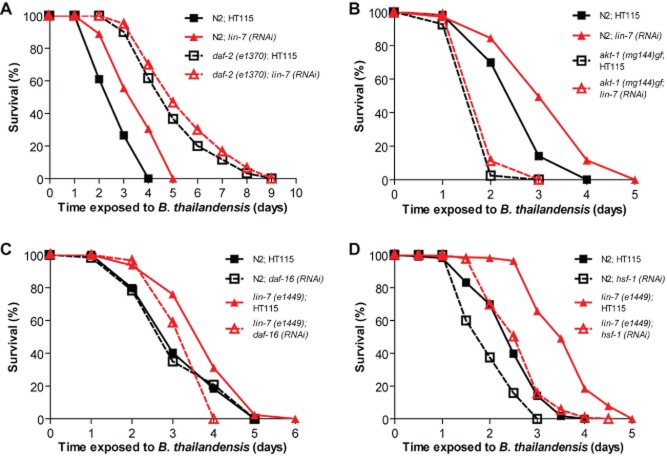
*lin-7* acts in the DAF-2 signalling pathway during infection. A. Wild-type or *daf-2 (e1370)* nematodes were first grown on parental *E. coli* HT115 before transferring to plates containing *B. thailandensis* ATCC (*P* < 0.0001). Wild-type (*P* < 0.0001) or *daf-2 (e1370)* (*P* = 0.1738; all *P* values as compared with its respective parental HT115) nematodes were also exposed to *lin-7* dsRNA before infection by *B. thailandensis* ATCC 700388. Due to the longer assay time, P_0_ nematodes were transferred to fresh plates containing *B. thailandensis* ATCC 700388 after every 2 days. B. Wild-type or *akt-1 (mg144)gf* nematodes were first grown on parental *E. coli* HT115 or exposed to *lin-7* dsRNA before transferring to plates containing *B. thailandensis* ATCC 700388. C. Wild-type (*P* = 0.8795) or *lin-7 (e1449)* (*P* < 0.0001; all *P* values as compared with its respective parental HT115) nematodes were first grown on parental *E. coli* HT115 or exposed to *daf-16* dsRNA before transferring to plates containing *B. thailandensis* ATCC 700388. D. Wild-type (*P* < 0.0001) or *lin-7 (e1449)* (*P* < 0.0001; all *P* values as compared with its respective parental HT115) nematodes were first grown on parental *E. coli* HT115 or exposed to *hsf-1* dsRNA before transferring to plates containing *B. thailandensis* ATCC 700388.

Based on the C-terminus binding motif information for the three proteins of the LIN-2/7/10 complex, we identified and tested five candidate receptors that have been shown or have the potential to interact with the LIN-2/7/10 complex (Fig. 3B). These included: INA-1, an alpha integrin subunit (Baum and Garriga, 1997); GPC-1 and GPC-2, both heterotrimeric guanine nucleotide-binding protein gamma subunits (Yamada *et al*., 2009); GLR-1, a glutamate receptor known to interact with LIN-10 at postsynaptic elements (Rongo *et al*., 1998); DAF-2, the insulin/IGF-1 RTK (Kenyon *et al*., 1993). We exposed nematodes carrying either loss-of-function mutations (*glr-1, gpc-1* and *daf-2*) or wild-type nematodes pre-treated with gene-specific dsRNA (*gpc-2* and *ina-1*) to *B. thailandensis* (Figs S3 and 4A)*.* Of the five receptors tested, only *daf-2 (e1370)* mutants survived significantly longer than wild-type nematodes upon *B. thailandensis* infection (*P* < 0.0001, Fig. 4A).

The DAF-2 RTK has a P-L-V motif at its extreme C-terminus (amino acids 1841–1843), raising the possibility that the DAF-2 C-terminus may potentially bind to the LIN-2 PDZ domain. To experimentally test this *in silico* prediction, we used the yeast two-hybrid system (Fields and Song, 1989). In this assay, the PDZ-containing portion of LIN-2 (amino acids 288–647 fused to the GAL4 transcriptional activation domain) interacted with the C-terminus of DAF-2 (amino acids 1445–1843 fused to the GAL4 DNA-binding domain), as detected by expression of both *ade2* and *his3* reporter genes downstream of two different GAL4-responsive promoters (Fig. 3C). Confirming the specificity of this interaction, the LIN-2 PDZ domain did not interact with human lamin C nor murine p53, and the DAF-2 C-terminus did not interact with SV40 large T antigen (Fig. 3C). These data suggest that LIN-7 binds to DAF-2 RTK via LIN-2, potentially regulating DAF-2 signalling activities.

### LIN-7 interacts with the DAF-2 insulin/IGF-1 signalling pathway

The findings that *daf-2 (e1370)* mutants exhibited enhanced survival when exposed to *B. thailandensis* and that the LIN-2 PDZ domain bound to the DAF-2 C-terminus suggested that the LIN-2/7/10 complex could potentially modulate the DAF-2 signalling pathway. We thus tested the extent to which perturbations in DAF-2 signalling might affect the *lin-7*-mediated infection phenotype. Upon *B. thailandensis* infection, *daf-2 (e1370)* mutants died at highly similar rates irrespective of *lin-7* status (*P* = 0.1738, Fig. 4A). This is consistent with the idea that *lin-7* and *daf-2* are acting in the same pathway and that *lin-7* may be acting upstream of *daf-2* as no additional survival advantage is imparted by *lin-7* dsRNA when *daf-2* is mutated.

If the *lin-7-*mediated infection phenotype involves DAF-2 signalling, then the enhanced survival exhibited by *lin-7* mutants should also be abolished by constitutively activating AKT-1, the PI3K kinase downstream of DAF-2 (Paradis and Ruvkun, 1998). Indeed, *akt-1 (mg144)* mutants carrying gain-of-function mutations in the PI3K kinase suppressed the infection phenotype conferred by *lin-7* RNAi treatment (*P* < 0.0001, Fig. 4B). This provides additional evidence that *lin-7* acts in the *daf-2* signalling pathway.

DAF-16 plays a pivotal role in phenotypes mediated by the DAF-2 signalling pathway; if LIN-7 acts in the same pathway as DAF-2 during infection, DAF-16 should also affect the survival ability of *lin-7* mutants. To assess the contribution of DAF-16, we compared the infection outcomes of wild-type nematodes and *lin-7* mutants in the presence or absence of *daf-16* dsRNA*.* Previous studies have shown that in wild-type nematodes, inhibiting *daf-16* alone is not sufficient to modulate survival during bacterial infection (Garsin *et al*., 2003; Evans *et al*., 2008). Our experimental results were consistent with these previous findings as we also found that *daf-16* dsRNA did not alter the survival of infected wild-type nematodes (*P* = 0.8795, Fig. 4C). In contrast, *daf-16* RNAi treatment significantly suppressed the enhanced survival exhibited by *lin-7* mutants (*P* < 0.0001, Fig. 4C), similar to that observed with *daf-2* mutants (Garsin *et al*., 2003). Collectively, these results suggest that the infection phenotype mediated by loss of *lin-7* activity is likely to be, at least in part, dependent on *daf-16* activity, supporting a role for *lin-7* in positively regulating *daf-2* signalling.

### *hsf-1* also contributes to the infection phenotype of *lin-7* mutants

In addition to DAF-16, HSF-1 is required for the enhanced survival of *daf-2* mutants during bacterial infection (Singh and Aballay, 2006). Together, HSF-1 and DAF-16 co-regulate certain subsets of genes, including the small HSPs (Hsu *et al*., 2003); these small HSPs were also found to be upregulated in *daf-2* mutants (Hsu *et al*., 2003; McElwee *et al*., 2003). Recent studies have furthermore revealed that, analogous to DAF-2's role of inhibiting DAF-16, the insulin signalling pathway also compromises HSF-1 activity by directly regulating a protein complex which sequesters and suppresses HSF-1, further strengthening a functional interplay between the insulin signalling pathway and the heat shock response (Chiang *et al*., 2012).

To investigate the possible contribution of HSF-1 towards the enhanced survival exhibited by *lin-7* mutants during infection, we compared the infection outcomes of wild-type nematodes and *lin-7* mutants in the presence or absence of *hsf-1* dsRNA*.* Consistent with previous findings (Singh and Aballay, 2006), the survival of infected wild-type nematodes was significantly reduced by *hsf-1* dsRNA (*P* < 0.0001, Fig. 4D). Importantly, *hsf-1* RNAi treatment also significantly suppressed the enhanced survival of *lin-7* mutants (*P* < 0.0001, Fig. 4D). These results suggest that the infection phenotype exhibited by *lin-7* mutants could also be, at least in part, dependent on *hsf-1* activity.

### *lin-7* affects DAF-16 nuclear localization upon heat shock

HSF-1 and one of its downstream HSPs, HSP-1, have been shown to affect the kinetics of DAF-16 nuclear localization in *C. elegans*; nematodes treated with *hsf-1* or *hsp-1* dsRNA exhibited delayed DAF-16::GFP nuclear egression upon heat shock (Singh and Aballay, 2009). These prior observations and the finding that loss of *hsf-1* activity suppressed the infection phenotype of *lin-7* mutants led us to investigate whether *lin-7* deficiency would also affect the kinetics of DAF-16 nuclear localization.

**Fig. 5:**
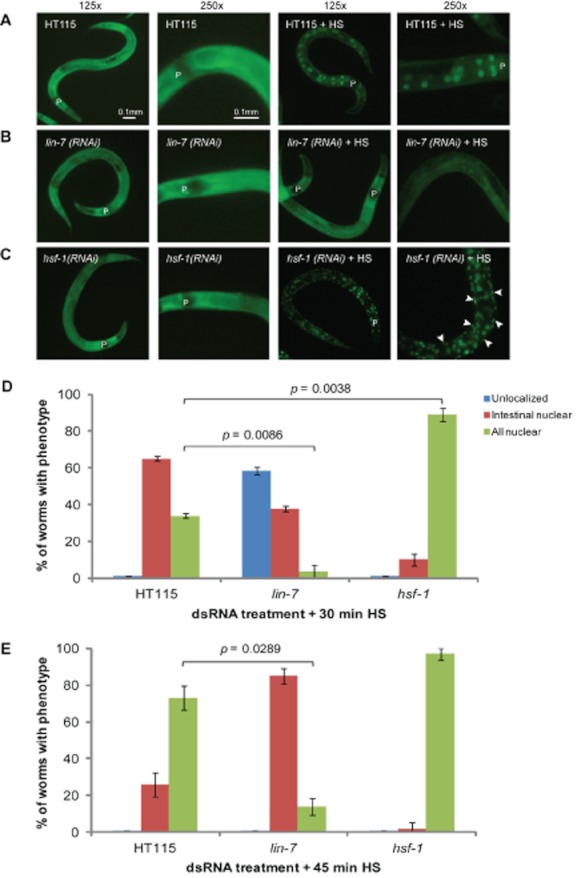
During acute heat stress, *lin-7* modulates the kinetics of DAF-16::GFP nuclear localization. A–C. DAF-16::GFP mutants were first grown on parental *E. coli* HT115 or exposed to *lin-7* or *hsf-1* dsRNA before subjecting to heat shock at 35°C in a water bath. Nematodes were harvested every 15 min and assayed before and immediately after heat shock (+ HS). Representative images after 30 min of heat shock are shown at 125× (first and third panels) or 250× magnification (second and fourth panels). The pharynx (P) and non-intestinal nuclei (white arrowheads) are indicated. Scale bar represents 0.1 mm. Images are representative of at least 50 nematodes per RNAi treatment from three independent assays. D and E. Nematodes were classified as exhibiting either uniformly distributed DAF-16::GFP (unlocalized), nuclear DAF-16::GFP in intestinal cells only (intestinal nuclear) or nuclear DAF-16::GFP in all cell types (all nuclear). The number of nematodes in each category was counted and shown as a percentage of total nematodes assayed immediately after acute heat stress of 30 min (D) or 45 min (E). Percentages that were significantly different from the parental HT115 treatment for the ‘all nuclear’ phenotype are indicated.

To study this, we used nematodes containing a DAF-16::GFP translational fusion protein to compare DAF-16 nuclear localization patterns under three different genetic backgrounds: no RNAi (parental HT115), *lin-7* RNAi and *hsf-1* RNAi. Consistent with previous reports (Henderson and Johnson, 2001), under non-stressed conditions (Brenner, 1974), DAF-16::GFP in all the three genetic backgrounds remained diffusely present throughout the nucleus and the cytoplasm in all tissues (‘unlocalized’; Fig. 5A–C, first and second panels). In our study, *B. thailandensis* infection alone was insufficient to effect any change in DAF-16::GFP nuclear localization in these nematodes (data not shown). Thus to assess the influence of *lin-7* on DAF-16 nuclear localization, we implemented a heat shock regimen at 35°C for 30 min, previously shown to induce DAF-16::GFP nuclear localization (Henderson and Johnson, 2001). Following the acute heat stress, nematodes treated with parental HT115 exhibited DAF-16::GFP nuclear localization predominantly restricted to the 28–32 nuclei of the intestinal epithelial cells (‘intestinal nuclear’; Fig. 5A, third and fourth panels); while the majority of *hsf-1* dsRNA-treated nematodes displayed DAF-16::GFP nuclear localization across all examined cell types, including the intestine and the hypodermis (‘all nuclear’; Fig. 5C, third and fourth panels). The number of *hsf-1* dsRNA-treated nematodes exhibiting the ‘all nuclear’ DAF-16::GFP localization pattern was significantly higher than those without RNAi treatment (*P* = 0.0038, Fig. 5D), consistent with previous findings that *hsf-1* dsRNA delayed DAF-16::GFP nuclear export (Singh and Aballay, 2009).

In contrast, *lin-7* dsRNA-treated nematodes exhibited slower DAF-16::GFP nuclear localization kinetics when compared with those without RNAi; upon 30 min of heat shock, *lin-7* dsRNA-treated nematodes predominantly exhibited ‘unlocalized’ DAF-16::GFP localization patterns (Fig. 5B, third and fourth panels; *P* = 0.0086, Fig. 5D). When the heat shock treatment was extended for another 15 min, nematodes treated with *lin-7* dsRNA predominantly exhibited the ‘intestinal nuclear’ DAF-16::GFP localization pattern when compared with control (*P* = 0.0289, Fig. 5E). It is worth noting that at this time point, nuclear localization of DAF-16::GFP appeared to be delayed in the hypodermis of *lin-7* dsRNA-treated nematodes. Further on, when heat shock was prolonged to 1 h, 100% of *lin-7* dsRNA-treated nematodes eventually revealed an ‘all nuclear’ DAF-16::GFP localization pattern, identical to nematodes treated with parental HT115 or *hsf-1* dsRNA (data not shown). These data show that *lin-7* can significantly affect the kinetics of DAF-16 nuclear localization during heat shock, further corroborating a connection between LIN-7 and the DAF-2 signalling pathway. In addition, these results raised the possibility that perhaps the increased nuclear export of DAF-16::GFP in *lin-7* dsRNA-treated nematodes could be attributed to *hsf-1*, given that reducing *hsf-1* activity suppressed this phenomenon. Henceforth, our observations also suggest a functional relationship between LIN-7, DAF-2 and HSF-1.

### Tissue-specific RNAi assays indicate hypodermal *lin-7* regulates infection outcomes

Although our initial RNAi screen focused on genes expressed in the nematode intestine, *lin-7* is known to function in non-intestinal tissues as well (Simske *et al*., 1996; Kaech *et al*., 1998). Various tissues in the nematode can also exhibit distinct aspects of pathogen defence (Pujol *et al*., 2008; Ren *et al*., 2009; Ziegler *et al*., 2009). These previous observations, coupled with the finding that *lin-7* seems to affect nuclear localization of DAF-16::GFP in the hypodermis upon 45 min of heat stress (Fig. 5E), led us to investigate the roles of intestinal and hypodermal *lin-7* in mediating the observed infection phenotype.

**Fig. 6:**
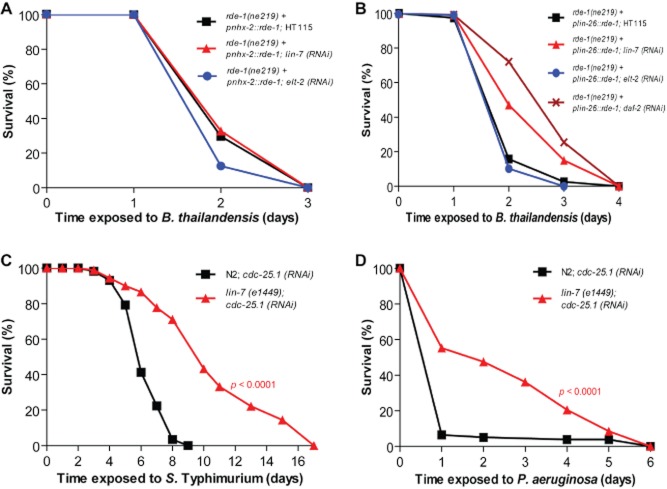
*lin-7* is a general host factor and functions specifically in the hypodermis during infection. A. *rde-1 (ne219)* mutants carrying the intestinal *pnhx-2::rde-1* transgene were first grown on parental *E. coli* HT115 or exposed to *lin-7* (*P* = 0.7298) or *elt-2* (*P* = 0.0140; all *P* values as compared with parental HT115) dsRNA before transferring to plates containing *B. thailandensis* ATCC 700388. B. *rde-1 (ne219)* mutants carrying the hypodermal *plin-26::rde-1* transgene were first grown on parental *E. coli* HT115 or exposed to *lin-7* (*P* < 0.0001), *daf-2* (*P* < 0.0001) or *elt-2* (*P* = 0.2679; all *P* values as compared with parental HT115) dsRNA before transferring to plates containing *B. thailandensis* ATCC 700388. C. Wild-type or *lin-7 (e1449)* nematodes were first exposed to *cdc-25.1* dsRNA before transferring to NGM plates containing *S.* Typhimurium ATCC 14028 (*P* < 0.0001). D. Wild-type or *lin-7 (e1449)* nematodes were first exposed to *cdc-25.1* dsRNA before transferring to peptone-glucose-sorbitol plates containing *P. aeruginosa* PA14 (*P* < 0.0001).

To study this, we performed tissue-specific RNAi experiments, specifically silencing *lin-*7 either in the intestine or in the hypodermis. Tissue-specific RNAi was achieved by feeding *lin-7* dsRNA to *rde-1 (ne219)* mutants carrying the wild-type *rde-1* transgene expressed either under the intestine-specific promoter *pnhx-2* (Espelt *et al*., 2005) or under the hypodermis-specific promoter *plin-26* (Qadota *et al*., 2007). *rde-1* encodes a member of the Argonaute protein family, whose expression is necessary to initiate RNAi in a cell-autonomous manner (Tabara *et al*., 1999). Tissue specificity of RNAi in these strains was confirmed by feeding them with *unc-22* dsRNA. As *unc-22* expression is restricted to the muscles (Moerman *et al*., 1988), neither strain showed the characteristic *unc-22* twitching phenotype as seen in wild-type nematodes (data not shown). As a further control for tissue specificity, we also treated these *rde-1 (ne219)* mutants with *elt-2* dsRNA. Consistent with reports that *elt-2* is specifically expressed in the intestine and protects against several bacterial pathogens (Fukushige *et al*., 1998; Kerry *et al*., 2006), we found that *elt-2* RNAi rendered *rde-1 (ne219)* mutants carrying the intestinal *pnhx-2::rde-1* transgene hypersensitive to *B. thailandensis* (*P* = 0.0140, Fig. 6A) but did not have an effect on *rde-1 (ne219)* mutants carrying the hypodermal *plin-26::rde-1* transgene (*P* = 0.2679, Fig. 6B).

Importantly, upon exposure to *B. thailandensis, rde-1 (ne219)* mutants carrying the hypodermal *plin-26::rde-1* transgene exhibited enhanced survival when treated with *lin-7* dsRNA (*P* < 0.0001, Fig. 6B). In contrast, treating *rde-1 (ne219)* mutants carrying the intestinal *pnhx-2::rde-1* transgene with *lin-7* dsRNA did not confer protection during infection (*P* = 0.7298, Fig. 6A), indicating that the *lin-7*-mediated infection phenotype probably involves mainly hypodermal and not intestinal *lin-7*. Interestingly, when treated with *daf-2* dsRNA, *rde-1 (ne219)* mutants carrying the hypodermal *plin-26::rde-1* transgene also exhibited enhanced survival upon *B. thailandensis* infection (*P* < 0.0001, Fig. 6B). This result provides further evidence for *lin-7* 's role in regulating *daf-2* signalling during infection and highlights the spatial specificity of this functional relationship in the hypodermis.

### Loss of *lin-7* function is protective against multiple bacterial pathogens

It has been previously shown that the DAF-2 insulin signalling pathway, as well as HSF-1, protects the nematode against multiple bacterial pathogens such as *Enterococcus faecalis*, *Pseudomonas aeruginosa* (Garsin *et al*., 2003; Singh and Aballay, 2006), and *Salmonella enterica* serovar Typhimurium (Singh and Aballay, 2006; Jia *et al*., 2009). If *lin-7* acts in the same pathway as *daf-2*, then *lin-7* mutants should also possess the ability to survive infections by other bacterial pathogens and not just specifically by *B. thailandensis*. To test this possibility, we exposed wild-type nematodes and *lin-7* mutants to two other bacterial pathogens, *S.* Typhimurium and *P. aeruginosa*, which are known to kill *C. elegans* by different mechanisms. *S.* Typhimurium kills *C. elegans* by slow intestinal colonization on NGM agar (Aballay *et al*., 2000; Labrousse *et al*., 2000), whereas *P. aeruginosa* produces a fast-killing nematocidal toxin on high osmolarity peptone-glucose-sorbitol agar (Mahajan-Miklos *et al*., 1999). We found that *lin-7* mutants consistently survived longer upon infection with either pathogen (*P* < 0.0001, Fig. 6C and D). These results establish *lin-7* as a general infection factor, consistent with the hypothesis that *lin-7* regulates *daf-2* signalling in *C. elegans*.

## Discussion

In this study, we identified a novel role for LIN-7 during bacterial infection. Loss of *lin-7* function imparted *C. elegans* the enhanced ability to survive infection by three distinct Gram-negative bacterial pathogens (*B. thailandensis, P. aeruginosa* and *S.* Typhimurium). Subsequent genetic and biochemical assays linked LIN-7 to DAF-2, a major regulator of the insulin/IGF-1 signalling pathway. Tissue-specific RNAi experiments revealed that *lin-7* likely functions in the hypodermal cells of *C. elegans* to affect infection outcomes. Collectively, our results suggest that during infection, LIN-7 regulates the DAF-2 signalling pathway in nematode hypodermal tissues and this modulation of DAF-2 signalling becomes detrimental to the infected host.

Infection sensitivity in *C. elegans* can be modulated by the removal of germline signals (Miyata *et al*., 2008) and by mutations that increase overall nematode lifespan (Garsin *et al*., 2003). Our results suggest that the infection phenotype of *lin-7* mutants is unlikely to be due to these two confounding factors. Specifically, *lin-7* loss-of-function mutants are not known to be germline-deficient and are well characterized for their matricidal phenotype (Horvitz and Sulston, 1980; Ferguson and Horvitz, 1985). In addition, when exposed to *B. thailandensis*, fertile *lin-7 (RNAi)* nematodes, fertile *lin-7 (e1449)* mutants as well as sterile *lin-7 (e1449)* mutants [via *cdc-25.1* RNAi (Evans *et al*., 2008; Shapira and Tan, 2008)] consistently exhibited enhanced survival over wild-type nematodes, indicating that the *lin-7*-mediated infection phenotype is not related to germline signalling. Furthermore, the lifespans of *lin-7* mutants and wild-type nematodes when grown on non-pathogenic *E. coli* were indistinguishable, suggesting that the infection phenotype exhibited by *lin-7* mutants is not simply a consequence of aberrant organismal development. These results thus support a more direct role for *lin-7* in influencing infection outcomes.

LIN-7 is known to associate in a tripartite complex with LIN-2 and LIN-10 to positively regulate the subcellular localization and activity of various signalling proteins (Kaech *et al*., 1998; Kim and Sheng, 2004; Alewine *et al*., 2007). Similar to *lin-7* mutants, *lin-2* and *lin-10* mutants also exhibited enhanced survival upon *B. thailandensis* infection. Our subsequent *in silico* analysis to identify potential interacting partners of the LIN-2/7/10 complex highlighted the DAF-2 RTK as a potential LIN-2 binding partner. Using the yeast two-hybrid system, we were able to confirm that the LIN-2 PDZ domain can indeed bind to the DAF-2 C-terminus. Along with genetic studies perturbing the insulin signalling pathway, our results suggest that LIN-7 binds, via LIN-2, to the DAF-2 C-terminus and may positively regulate DAF-2 signalling. Still, from our study we cannot exclude the possibility that during infection, LIN-7 can also bind and regulate hitherto unidentified proteins.

Our data suggest that the modulation of DAF-2 signalling by LIN-7 occurs predominantly in the *C. elegans* hypodermis. Interestingly, preceding research has implicated the hypodermis as an important tissue for influencing infection outcomes. For example, infection by the fungus *Drechmeria coniospora* activates signalling pathways in the hypodermis that in turn initiates an intestinal immune response (Pujol *et al*., 2008). In addition, intestinal *E. faecalis* infection has been shown to activate host NADPH oxidases to generate ROS in both the hypodermis and the intestine as a protective immune measure (Chávez *et al*., 2009). Thus, even though the host–pathogen interface is primarily localized to the intestine, tissues outside the intestine (such as the hypodermis) clearly can also contribute to the overall survival outcome of an infection.

DAF-16 is well established to positively regulate immune genes including lysozymes, catalases, saposins and superoxide dismutases (Murphy *et al*., 2003). The finding that inhibition of *hsf-1* suppressed the infection phenotype exhibited by *lin-7* mutants suggested that in addition to DAF-16, HSF-1 also plays a significant role in the ability of LIN-7 to modulate host infection outcomes. This is consistent with the findings that (i) LIN-7 and DAF-2 act in the same pathway (this study), and (ii) both DAF-16 and HSF-1 suppressed the enhanced survival of *daf-2* mutants (Garsin *et al*., 2003; Singh and Aballay, 2006).

Although the exact interaction between the insulin signalling pathway and HSF-1 during infection is not yet well characterized, recently, this interplay has been dissected in the context of ageing. DAF-2 insulin signalling inhibits DDL-1 phosphorylation, thus allowing DDL-1 and DDL-2 to form a complex sequestering and inhibiting HSF-1 (Chiang *et al*., 2012). Hence, in a *daf-2* mutant, HSF-1 is not sequestered by DDL-1/2. Considering these observations, our data suggest that LIN-7 may positively regulate DAF-2 signalling and hence inhibit HSF-1 activity. This view is supported by the findings that (i) the infection phenotype of *lin-7* mutants was suppressed when treated with *hsf-1* dsRNA, and (ii) loss of *lin-7* resulted in a significant delay in DAF-16::GFP nuclear localization.

HSF-1 and its downstream HSPs have been shown to protect the infected host by reducing protein aggregation detrimental to host cellular tissues. This undesired protein aggregation has been proposed to be caused by reactive oxygen species generated in the nematode in response to infection (Mohri-Shiomi and Garsin, 2008). The inhibitory function of DAF-2 on HSF-1 activity may thus provide a possible explanation for the reduction in protein aggregation observed in *daf-2* mutants (Chávez *et al*., 2009). Similarly, in *lin-7* mutants, this protective pathway could also have been enhanced to counter infection-induced protein aggregation. This further hints at the possibility that *lin-7* mutants, instead of being resistant (which we have shown earlier on not to be the case), could be tolerant of infection, restricting the damage inflicted on host tissues (Schneider and Ayres, 2008).

Another possible explanation for the infection phenotype exhibited by *lin-*7 mutants revolves around the fact that throughout evolution from nematodes to mammals, it is absolutely crucial to have a tight surveillance over host defence pathways as both insufficient and excessive activity can prove detrimental. Although DAF-16 is known to positively regulate immune-related genes (Murphy *et al*., 2003), excessive DAF-16 transcriptional activity can also result in enhanced susceptibility to bacterial infections (Singh and Aballay, 2009). In that study, it was shown that nematodes with additional gene copies of *daf-16*, coupled with either acute heat stress or *daf-2* loss-of-function mutations, actually exhibited an increased rather than reduced susceptibility to bacterial infections, indicating that while DAF-16 is essentially protective during infection, excessive DAF-16 nuclear activity could also be detrimental rather than beneficial.

At present it remains unclear exactly why excessive DAF-16 nuclear accumulation is detrimental. It is possible that DAF-16 hyperactivation upregulates AQP-1, a membrane water channel protein, and this results in dysregulated water homeostasis, host cellular damage and reduced immune responses (Singh and Aballay, 2009). It is thus not far-fetched to speculate why DAF-2 signalling also has an inhibitory effect on HSF-1. When *daf-2* mutants are exposed to bacterial pathogens, excessive DAF-16 nuclear accumulation may occur due to lack of DAF-16 phosphorylation by upstream PI3K kinases. However, along with an enhanced HSF-1 activity which promotes DAF-16 nuclear export (Singh and Aballay, 2009), *daf-2* mutants would be able to maintain a homeostatic check on DAF-16 nuclear activity. In the same way, *lin-7* mutants may have the ability to maintain advantageous levels of DAF-16 in the hypodermal tissues and this, in part, confers protection to the whole nematode.

In summary, our findings revealed a role for the *C. elegans* cell junction protein, LIN-7, in the modulation of hypodermal DAF-2 signalling and hence host infection outcomes. Interestingly, several other *C. elegans* genes functioning in early developmental or physiological processes also appear to have been reused in the adult nematode for pathogen defence (Lin *et al*., 1997; Honda and Honda, 1999; Irazoqui *et al*., 2010). In vertebrates, LIN-7 homologues are expressed ubiquitously in various tissues, including the junctional complex regions of kidney cells (Irie *et al*., 1999), synaptic junctions in neurones (Butz *et al*., 1998) and basolateral membranes of epithelial cells (Yan *et al*., 2009). The LIN-2/7/10 complex is also conserved from *C. elegans* to mammals: mammalian LIN-7 interacts with the LIN-2 homologue CASK (Cohen *et al*., 1998) and the LIN-10 homologue X11/Mint (Rongo *et al*., 1998) to establish cell junctions in epithelial cells and synapses. Given that some pathogen defence pathways are evolutionarily conserved from invertebrates to mammals, it is intriguing to speculate that perhaps this is one of the earlier functions of LIN-7 and that during evolution, LIN-7 homologues have acquired additional roles to cope with more complex systems and processes in higher organisms.

## Experimental procedures

### Nematode, bacteria and yeast strains

Nematode, bacteria and yeast strains used in this study are listed in Table S1. All nematode strains were cultured and maintained at 20°C on modified nematode growth media (NGM, 0.35% peptone) agar and fed with *E. coli* strain OP50, as described (Brenner, 1974). Except for *daf-2 (e1370)* mutants, this strain was cultured and maintained at 15°C to suppress dauer formation. Bacteria strains were grown in Luria–Bertani (LB) broth at 37°C. Yeast strains were grown in yeast extract peptone dextrose (YPD) broth at 30°C.

### Survival assays

*Burkholderia thailandensis* ATCC 700388 and E555, *P. aeruginosa* PA14 and *S.* Typhimurium ATCC 14028 were grown overnight in LB broth at 37°C. *B. thailandensis* and *S.* Typhimurium lawns were prepared by spreading 100 μl of overnight culture on modified NGM agar and grown for 24 h at 37°C. *P. aeruginosa* lawns were prepared similarly by spreading on peptone-glucose-sorbitol agar (Mahajan-Miklos *et al*., 1999). Unless specified otherwise, 40 L4-staged nematodes were added to each lawn and infected as per described (Powell and Ausubel, 2008). Nematodes were set down on bare agar before transferring to pathogen-containing lawns to minimize the transfer of *E. coli.* No visible *E. coli* growth on pathogen-containing lawns was observed at locations where nematodes were added nor was there any crowding of nematodes at such locations. To further test for *E. coli* contamination, nematodes were removed 24 h post infection; pathogen-containing lawns were harvested, diluted appropriately in M9 buffer (Brenner, 1974) and tested for *E. coli* and *B. thailandensis* by plating on neat LB agar and LB agar supplemented with gentamicin 25 μg ml^−1^ (Thermo Fisher Scientific Waltham, MA)*.* No *E. coli* contamination on pathogen-containing lawns was observed (Fig. S4). Nematode survival was scored at 24°C and nematodes were considered dead upon failure to respond to gentle touch by a platinum wire. Results are representative of three independent experiments.

### Immunofluorescence assays

Nematodes infected by *B. thailandensis* E555 were prepared for immunohistochemical staining using a freeze-crack method (Duerr *et al*., 1999) and fixed using 50% methanol (2 min) and 50% acetone (4 min). After washing, slides were blocked for 1 h in 5% bovine serum albumin (BSA) in antibody buffer [0.5% Triton X-100, 1 mM EDTA, 0.1% BSA and 0.05% sodium azide (NaN_3_) in phosphate-buffered saline (PBS)], followed by 1 h primary antibody incubations using monoclonal antibody 3015 IgG1 (Sim *et al*., 2010). Secondary antibody incubations were performed using donkey anti-mouse Texas Red (1:500) (Jackson ImmunoResearch, West Grove, PA) for 4 h. All incubations were performed at 24°C. Slides were mounted in anti-photobleaching media with DAPI (Vector Laboratories, Burlingame, CA) and visualized on a LEICA DMRE microscope. Images were analysed by GNU Image Manipulation Program (version 2.6.3). For GFP experiments, nematodes fed on *E. coli* OP50::GFP lawns were harvested and mounted for microscopy in PBS with 25 mM NaN_3_. Nematodes were visualized on a Carl Zeiss Axiovert 200m inverted microscope and images were analysed by Metamorph software (version 6.3r7). Images are representative of at least 50 nematodes from three independent assays.

### RNAi assays

Unless specified otherwise, RNAi assays were carried out at 20°C by feeding nematodes with parental *E. coli* HT115 (DE3) strain or *E. coli* HT115 clones expressing gene-specific dsRNA (Timmons and Fire, 1998). Each clone identity was verified by direct sequencing using specific oligonucleotides targeting the L4440 vector: pL4440-F (gTTTTCCCAgTCACgACgTT) and pL4440-R (TggATAACCgTATTACCgCC) (Rual *et al*., 2004). RNAi assays were performed by growing each clone for 8 h in LB broth supplemented with ampicillin 50 μg ml^−1^ (Sigma-Aldrich, St. Louis, MO) and seeding on isopropyl β-d-1-thiogalactopyranoside (IPTG)-containing modified NGM agar. Nematode embryos, generated by hypochlorite treatment, were propagated on these seeded plates until the L4 stage. Nematodes were subsequently transferred to pathogen-containing lawns.

For experiments involving sterile nematodes, embryos were exposed to *cdc-25.1* dsRNA at late embryogenesis till day 1 adult stage, before transferring them to pathogen-containing lawns. Under such conditions, *cdc-25.1* RNAi resulted in nematodes with an Emb phenotype (Evans *et al*., 2008; Shapira and Tan, 2008).

For experiments involving *hsf-1* RNAi, nematode embryos were exposed to *hsf-1* dsRNA at 15°C until the L4 stage, before transferring them to pathogen-containing lawns. Under such conditions, *hsf-1* RNAi did not result in larva developmental arrest observed when the assay was carried out at higher temperatures (Walker *et al*., 2003; Singh and Aballay, 2006).

### Nematode bacterial load analysis

Nematodes were infected with *B. thailandensis* ATCC 700388 as per described. At 24 h post infection, infected nematodes were harvested and set down on bare agar before transferring to M9 buffer to minimize the contamination of bacteria*.* Nematodes were washed thrice with M9 buffer, followed by 1 h incubation in M9 buffer containing trypsin-EDTA (Life Technologies, Carlsbad, CA) to remove bacteria present on the exterior of the nematode. Nematodes were then washed thrice with M9 buffer only to remove trypsin-EDTA, and subsequently lysed by vortexing with 400 g silicon-carbide sharp particles (Biospec, Bartlesville, OK) and 0.2% sodium dodecyl sulfate. Lysates were diluted appropriately in M9 buffer and plated on LB agar supplemented with gentamicin to select for *B. thailandensis*. After 2-day incubation at 37°C, amount of live bacteria per nematode was determined by cfu counts. At least 25 nematodes were harvested per nematode strain and experiments were performed in triplicates.

### Yeast two-hybrid assays

Plasmids and primers used in this assay are listed in Table S3. The PDZ domain of LIN-2 (amino acids 288–647) and the C-terminus of DAF-2 (amino acids 1445–1843) were PCR-cloned using template cDNA from a mixed population of wild-type N2 nematodes. PCR fragments were cloned, at EcoRI and BamHI restriction sites for *daf-2*, or at NdeI and XmaI sites for *lin-2*, in-frame into the GAL4 DNA-binding domain (DNA-BD) vector pGBKT7 or GAL4 activation domain (AD) vector pGADT7 (Clontech Laboratories, Mountain View, CA) respectively. Reagents provided by the manufacturer included: (i) positive controls – murine p53 and SV40 large T antigen, as fusion proteins with the same GAL4 DNA-BD and GAL4 AD, respectively, and (ii) negative control – human lamin C, fused to the GAL4 DNA-BD.

Y2HGold reporter and Y187 mating haploid yeast strains were transformed individually with either the GAL4 DNA-BD or AD construct, according to the manufacturer's instructions. Successfully transformed haploids were selected on plates lacking tryptophan or leucine accordingly. Six pairwise combinations of transformed Y2HGold and Y187 haploid strains (see Fig. 3C) were allowed to mate overnight in YPD broth and diploids were selected on synthetically defined media lacking leucine, tryptophan, adenine and histidine. Growth within 3 days of incubation at 30°C indicated positive protein–protein interactions within mated pairs. Clone identities were verified by extracting plasmids from yeast diploids harbouring positive LIN-2/DAF-2 interactions and rescuing them in *E. coli* TG1 strain, followed by direct sequencing of plasmids isolated from TG1 using the following pairs of oligonucleotides: daf2-check-F (TgACgATTCAgAAgCACTgg) and daf2-check-R (CATCTTgTCCACCACgTgTC); lin2-check-F (AgTggCAggTTTgACgAgAC) and lin2-check-R (AgTTgTCggAgT TCCAATgC).

### DAF-16 nuclear localization assays

Embryos from DAF-16::GFP nematodes, generated by hypochlorite treatment, were propagated on plates containing parental *E. coli* HT115 strain or *E. coli* HT115 clones expressing *lin-7* or *hsf-1* dsRNA. Nematodes were grown at 20°C for parental HT115 and *lin-7* dsRNA, or at 15°C for *hsf-1* dsRNA until the L4 stage. Acute heat shock was performed at 35°C for intervals of 15 min by placing sealed plates into a water bath. Nematodes before and immediately after heat shock were visualized using an Olympus MVX10 dissecting microscope under 125× or 250× total magnification and images were analysed by DP controller software (version 3.1.1.267). Nematodes were classified as exhibiting either uniformly distributed DAF-16::GFP (unlocalized), nuclear DAF-16::GFP restricted to the 28–32 nuclei of the intestinal epithelial cells only (intestinal nuclear) or nuclear DAF-16::GFP in all cell types (all nuclear). At least 50 nematodes were counted per RNAi treatment and experiments were performed in triplicates.

### Statistical analysis

Survival curves were analysed using the PRISM (version 5.0) software. Kaplan-Meier survival curves with *P* values < 0.05 were considered significantly different from the control. Student's *t*-test was used to analyse nematode bacteria loads and DAF-16::GFP nuclear localization results.

## Supporting information

**Fig. S1.** *B. thailandensis*-infected nematodes at 32 h post infection, related to Fig. 1. Wild-type nematodes were exposed to *B. thailandensis* strain E555 for 32 h. Infected nematodes were then fixed, permeabilized and labelled with monoclonal antibody 3015 and DAPI. In these merged images, *B. thailandensis* E555 is shown in red and DAPI in blue. Images are shown at 40× (left) or 100× magnification (right). The pharynx (P) and individual bacteria (white arrowheads) are indicated. Scale bar represents 0.01 mm. Images are representative of at least 50 nematodes from three independent assays.

**Fig. S2.** Empty RNAi vector L4440 did not affect infection sensitivity, related to Fig. 2. Wild-type nematodes were first grown on parental HT115 or HT115 with an empty RNAi vector L4440 before transferring to plates containing *B. thailandensis* ATCC 700388 (*P* = 0.2896). Each survival curve is representative of three independent experiments, each with three plates per strain and 40 nematodes per plate.

**Fig. S3.** Other potential interacting partners of the LIN-2/7/10 complex did not affect infection sensitivity, related to Fig. 3. A. Wild-type, *glr-1 (n2461)* (*P* = 0.3561) or *gpc-1 (pk298)* (*P* = 0.0908; all *P* values as compared with wild-type) nematodes were exposed to *B. thailandensis* ATCC 700388.

B. Wild-type nematodes were first grown on parental *E. coli* HT115 or exposed to *gpc-2* (*P* = 0.0590) or *ina-1* (*P* = 0.9832) dsRNA before transferring to plates containing *B. thailandensis* ATCC 700388.

**Fig. S4.** No *E. coli* contamination was observed on *B. thailandensis* pathogen lawns, related to *Experimental procedures*. Wild-type nematodes were exposed to *B. thailandensis* ATCC 700388. 24 h post infection, infected nematodes were removed. Pathogen-containing lawns were then harvested and plated on neat LB agar and LB agar with gentamicin to test for *E. coli* and *B. thailandensis*. No *E. coli* contamination was observed on infected *B. thailandensis* lawns (*P* = 0.7973). Results are representative of three independent experiments.

**Table S1.** List of nematode, bacteria and yeast strains used in this study, related to all figures.

**Table S2.** List of *C. elegans* intestinal genes chosen for initial RNAi screen, related to Fig. 2.

**Table S3.** List of plasmid constructs used in this study, related to all figures.
