# Supplementary material for: Modulation of Caenorhabditis elegans infection sensitivity by the LIN-7 cell junction protein
Source: Cell Microbiol. 2012 Jun 21;14(10):1584–99. doi: 10.1111/j.1462-5822.2012.01824.x (PMC3470699; doi:10.1111/j.1462-5822.2012.01824.x)
Supplement: Supplementary file 1 [file cmi0014-1584-SD1.pdf]

Modulation of *Caenorhabditis elegans* Infection Sensitivity by the LIN-7 Cell Junction Protein

(Supplemental Information)

XiaoHui Sem[1,2], Jason F. Kreisberg[1], Trupti Kawli[3], Man-Wah Tan[3,4,5], Mikael Rhen[2], Patrick Tan[1,6,7*]

[1]Genome Institute of Singapore, Singapore 138672, Republic of Singapore

[2]Department of Microbiology, Tumor and Cell Biology, Karolinska Institutet, Stockholm 17177, Sweden

[3]Department of Genetics, Stanford University School of Medicine, Stanford, CA 94305, United States of America

[4]Department of Microbiology and Immunology, Stanford University School of Medicine, Stanford, CA 94305, United States of America

[5]Department of Microbial Pathogenesis, Genentech, Inc., South San Francisco, CA 94099, United States of America

[6]Duke-NUS Graduate Medical School Singapore, Singapore 169547, Republic of Singapore

[7]Cancer Sciences Institute of Singapore, National University of Singapore, Singapore 117456, Republic of Singapore

*Corresponding author.

E-mail address: tanbop@gis.a-star.edu.sg.

Tel: 65-6808-8182

Fax: 65-6808-8306

Fig. S1. ***B. thailandensis*-infected nematodes at 32 h post-infection, related to Figure 1.**

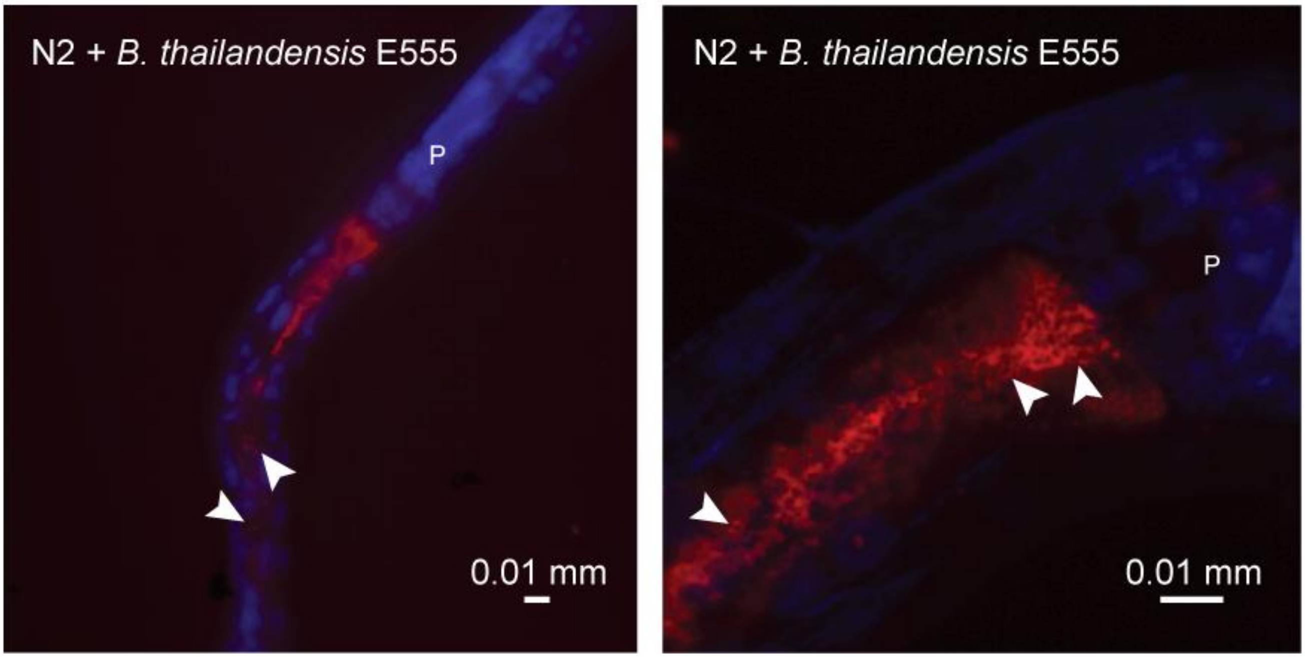

Wild-type nematodes were exposed to *B. thailandensis* strain E555 for 32 h. Infected nematodes were then fixed, permeabilized and labelled with monoclonal antibody 3015 and DAPI. In these merged images, *B. thailandensis* E555 is shown in red and DAPI in blue. Images are shown at 40x (left) or 100x magnification (right). The pharynx (P) and individual bacteria (white arrowheads) are indicated. Scale bar represents 0.01 mm. Images are representative of at least 50 nematodes from 3 independent assays.

Fig. S2. Empty RNAi vector L4440 did not affect infection sensitivity, related to Figure 2.

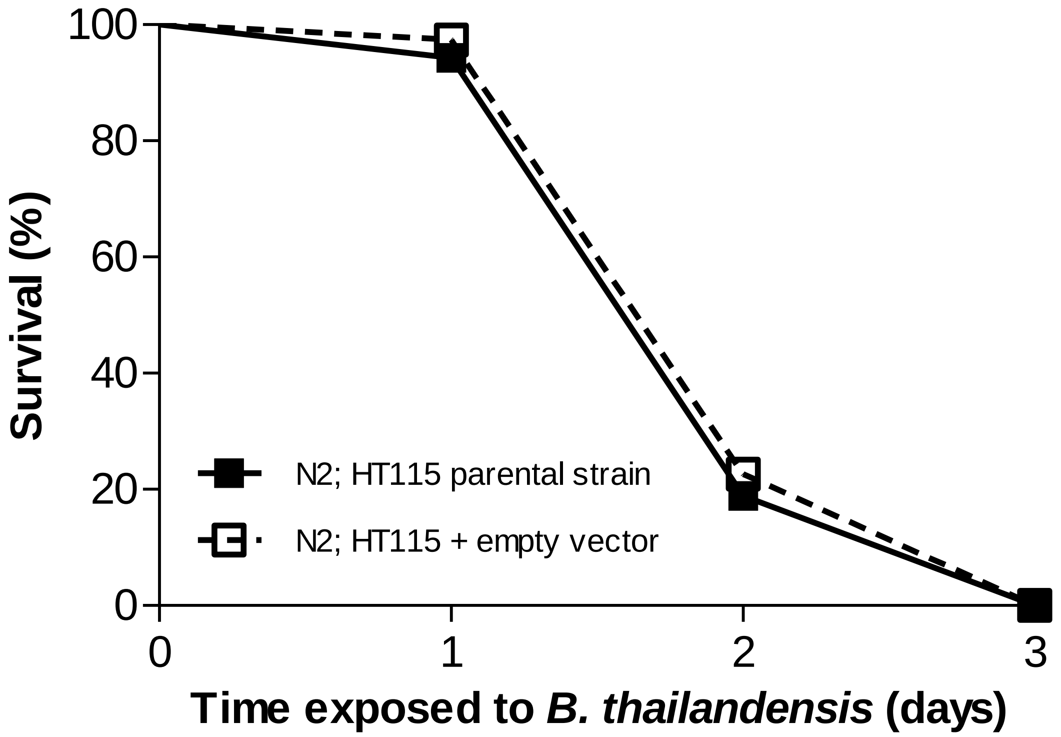

Wild-type nematodes were first grown on parental HT115 or HT115 with an empty RNAi vector L4440 before transferring to plates containing *B. thailandensis* ATCC 700388 (p = 0.2896). Each survival curve is representative of 3 independent experiments, each with 3 plates per strain and 40 nematodes per plate.

Fig. S3. Other potential interacting partners of the LIN-2/7/10 complex did not affect infection sensitivity, related to Figure 3.

A

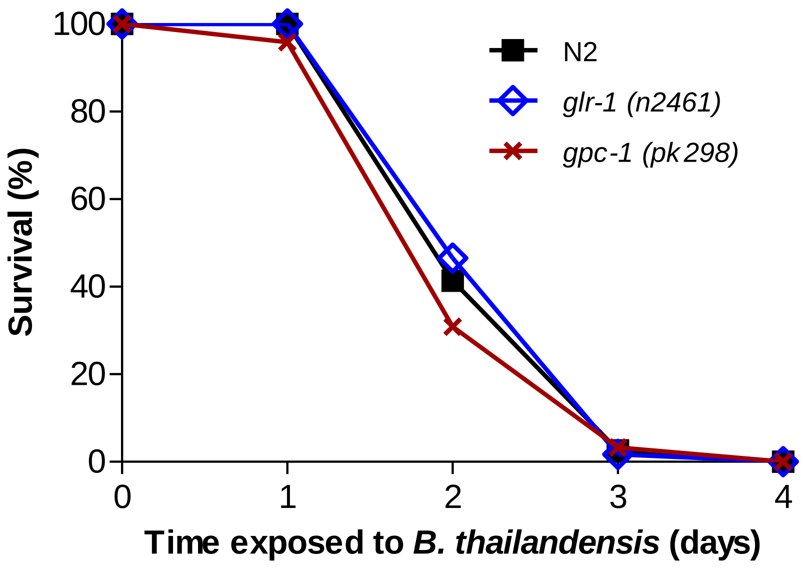

B

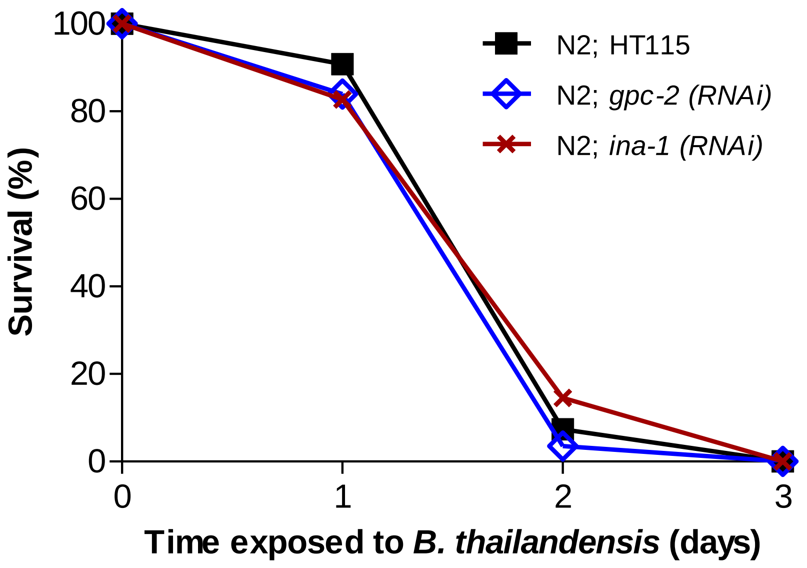

(A) Wild-type, *glr-1 (n2461)* (*p* = 0.3561) or *gpc-1 (pk298)* (*p* = 0.0908; all *p* values as compared to wild-type) nematodes were exposed to *B. thailandensis* ATCC 700388.

(B) Wild-type nematodes were first grown on parental *E. coli* HT115 or exposed to *gpc-2* (*p* = 0.0590) or *ina-1* (*p* = 0.9832) dsRNA before transferring to plates containing *B. thailandensis* ATCC 700388.

Fig. S4. No *E. coli* contamination was observed on *B. thailandensis* pathogen lawns, related to *Experimental procedures.*

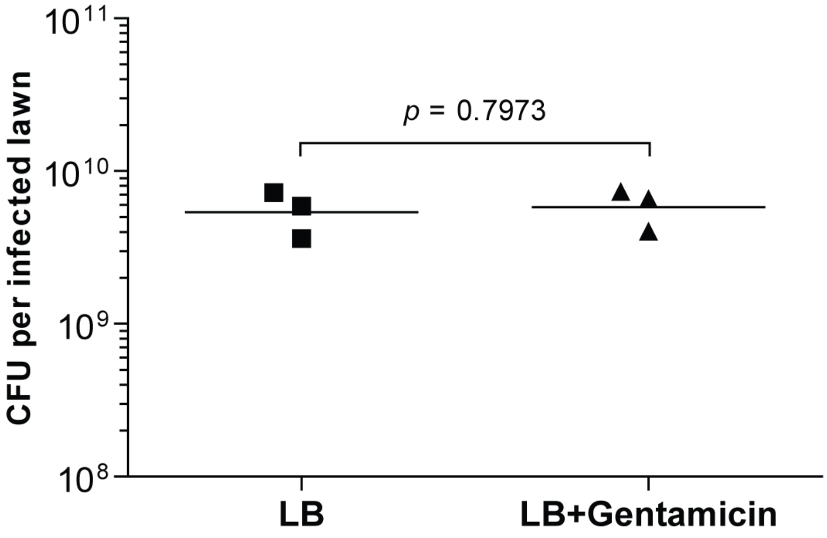

Wild-type nematodes were exposed to *B. thailandensis* ATCC 700388. 24 h post-infection, infected nematodes were removed. Pathogen-containing lawns were then harvested and plated on neat LB agar and LB agar with gentamicin to test for *E. coli* and *B. thailandensis*. No *E. coli* contamination was observed on infected *B. thailandensis* lawns ($p = 0.7973$). Results are representative of 3 independent experiments.

Supplemental Table S1, List of nematode, bacteria and yeast strains used in this study, related to all Figures.

	Strain	Genotype or description	Source or reference
**Nematode**			
C. elegans	N2	Wild-type variant Bristol	CGC[a]
	CB1449	*lin-7 (e1449)*	CGC[a]
	CB1413	*lin-7 (e1413)*	CGC[a]
	MT106	*lin-7 (n106)*	(Ferguson and Horvitz, 1985)
	CB1309	*lin-2 (e1309)*	CGC[a]
	MT3252	*lin-10 (n1402)*	CGC[a]
	PS21	*let-23 (sy1); him-5 (e1490)*	CGC[a]
	KP4	*glr-1 (n2461)*	CGC[a]
	NL792	*gpc-1 (pk298)*	CGC[a]
	CB1370	*daf-2 (e1370)*	CGC[a]
	GR1310	*akt-1 (mg144)gf*	CGC[a]
	TJ356	*daf-16::gfp [zls356 (pdaf-16::daf-16-gfp; rol-6)]*	CGC[a]
	NR222	*rde-1(ne219) V; kzIs9 [pKK1260(plin-26::nls::gfp) + pKK1253(plin-26::rde-1) + pRF6(rol-6(su1006)]*	(Kawli *et al.*, 2010; Qadota *et al.*, 2007)
	VP303	*rde-1(ne219); kbEx200 [rol-6(su1006);pnhx-2::rde-1]*	(Kawli *et al.*, 2010; Espelt *et al.*, 2005)
**Bacteria**			
E. coli	OP50	Laboratory food source	CGC[a]
	OP50::GFP	OP50 + pFVP25.1, ampicillin[r]	CGC[a]
	HT115 (DE3)	RNAi feeding parental strain, tetracycline[r]	Geneservice[b]
	HT115(DE3) / pL4440 (*lin-7*)	RNAi feeding clone targeting *lin-7*, ampicillin[r] tetracycline[r]	Geneservice[b]
	HT115 (DE3) / pL4440	RNAi feeding parental strain with empty pL4440 vector, ampicillin[r] tetracycline[r]	This study
	HT115(DE3) / pL4440 (*cdc-25.1*)	RNAi feeding clone targeting *cdc-25.1*, ampicillin[r] tetracycline[r]	Geneservice[b]
	HT115(DE3) / pL4440 (*gpc-2*)	RNAi feeding clone targeting *gpc-2*, ampicillin[r] tetracycline[r]	Geneservice[b]
	HT115(DE3) / pL4440 (*ina-1*)	RNAi feeding clone targeting *ina-1*, ampicillin[r] tetracycline[r]	Geneservice[b]
	HT115(DE3) / pL4440 (*daf-16*)	RNAi feeding clone targeting *daf-16*, ampicillin[r] tetracycline[r]	Geneservice[b]
	HT115(DE3) / pL4440 (*hsf-1*	RNAi feeding clone targeting *hsf-1*, ampicillin[r] tetracycline[r]	Geneservice[b]
	HT115(DE3) / pL4440 (*unc-22*)	RNAi feeding clone targeting *unc-22*, ampicillin[r] tetracycline[r]	Geneservice[b]

	HT115(DE3) / pL4440 (*elt-2*)	RNAi feeding clone targeting *elt-2*, ampicillin[r] tetracycline[r]	Geneservice[b]
	HT115(DE3) / pL4440 (*daf-2*)	RNAi feeding clone targeting *daf-2*, ampicillin[r] tetracycline[r]	(Chávez *et al.*, 2007)
	DH5α	Cloning strain for yeast two-hybrid	ATCC[c]
	TG1	Cloning strain for yeast two-hybrid	ATCC[c]
B. thailandensis	ATCC 700388	Reference strain isolated from Thailand, gentamicin[r]	ATCC[c]
	E555	Variant strain isolated from Cambodia, gentamicin[r]	(Sim *et al.*, 2010)
P. aeruginosa	PA14	Wild-type PA14 strain	Lian-Hui Zhang
S. Typhimurium	ATCC 14028	Wild-type 14028 strain	ATCC[c]
**Yeast**			
S. cerevisiae	Y2HGold	Reporter strain (MATa)	Clontech[d]
	Y187	Mating strain (MATα)	Clontech[d]
	Y2HGold / [pGBKT7-Daf2]	Reporter strain with DAF-2 C-terminus fused with GAL4 DNA-BD	This study
	Y187 / [pGADT7-Lin2]	Mating strain with LIN-2 PDZ domain fused with GAL4 AD	This study
	Y2HGold / [pGBKT7-53]	Reporter strain with murine p53 fused with GAL4 DNA-BD	This study
	Y2HGold / [pGBKT7-Lam]	Reporter strain with human lamin C fused with GAL4 DNA-BD	This study
	Y187 / [pGADT7-T]	Mating strain with SV40 large T antigen fused with GAL4 AD	This study

[a] Caenorhabditis Genetics Center, University of Minnesota, Minneapolis, U.S.A.

[b] Geneservice, Cambridge, U.K.

[c] American Type Cell Collection, Virginia, U.S.A.

[d] Clontech Laboratories Inc., California, U.S.A.

Supplemental Table S2, List of *C. elegans* intestinal genes chosen for initial RNAi screen, related to Figure 2.

Sequence name	Gene name	Gene description
F58E10.4	*aip-1*	AN-1-like zinc finger-containing protein
F31A9.3	*arg-1*	Delta/Serrate/LAG-2 family of transmembrane signaling ligands
C27A12.8	*ari-1*	predicted E3 ubiquitin ligase
C54D2.5	*cca-1*	calcium channel alpha subunit
F35E8.11	*cdr-1*	cadmium-inducible lysosomal family that affects susceptibility to cadmium toxicity
F25F2.2	*cdh-4*	homolog of the cadherin superfamily that is involved in cell-cell adhesion
Y47H9C.4	*ced-1*	transmembrane protein on phagocytic cells
E04F6.11	*clh-3*	chloride channel homologous to the mammalian channel ClC-2
C52E4.1	*cpr-1*	cysteine protease of the cathepsin B-like cysteine protease family
F11C7.4	*crb-1*	transmembrane protein with EGF-like and laminin repeats
Y38A10A.5	*crt-1*	ortholog of calreticulin (a calcium-binding molecular chaperone of the ER)
F33H1.1	*daf-19*	RFX family of transcription factors and is required for sensory neuron cilium formation
F56D1.5	*dhs-5*	predicted steroid dehydrogenase
K07E12.1	*dig-1*	giant member of the immunoglobulin superfamily
T12E12.4	*drp-1*	dynamin-related protein
C18B12.3	*dsc-1*	predicted gene, no known function
F47G6.1	*dyb-1*	homolog of mammalian alpha-dystrobrevin
F16B3.1	*egl-2*	voltage-gated potassium channel that affects egg laying, muscle activation, defecation, mechanosensation, and chemosensation
T22B7.1	*egl-13*	SOX domain transcription factor
H35N03.1	*exp-1*	excitatory, cation-selective GABA receptor
C01G8.5	*erm-1*	ortholog of the ERM family of cytoskeletal linkers
T01D1.2	*etr-1*	muscle-specific ELAV-type RNA-binding protein
F12F3.1	*exp-2*	six-transmembrane voltage-activated family of potassium channels
EGAP9.2	*fut-2*	alpha 1,2-fucosyltransferase specifically expressed in intestinal cells
ZC308.1	*gld-2*	catalytic subunit of a cytoplasmic poly(A) polymerase associated with P granules in early embryos
F22D6.11	*gly-18*	branching enzyme similar to N-acetylglucosaminyltransferase
M05B5.5	*hlh-2*	Class I basic helix-loop-helix transcription factor
C02B8.4	*hlh-8*	helix-loop-helix protein required for normal muscle development
M6.1	*ifc-2*	one of the three isoforms of an intermediate filament protein
R107.4	*ikke-1*	inhibitor of NFKappaB kinase epsilon subunit homolog
C36B1.12	*imp-1*	member of the intramembrane protease family
T22C1.7	*jph-1*	junctophilin implicated in the formation of the junctional membrane complex
T02G5.8	*kat-1*	homolog of the human gene ACAT1
T09A5.2	*klp-3*	C-terminal kinesin motor protein
Y54G11A.10	*lin-7*	cell junction protein which likely serves as an organizational center for large macromolecular complexes in polarized cells

R107.8	*lin-12*	Notch/LIN-12/glp-1 transmembrane receptor family
F18A1.3	*lir-1*	LIN-26-like zinc-finger protein that shares a unique C2H2 motif together with LIR-2, LIR-3, and LIN-26
Y22F5A.4	*lys-1*	putative lysozyme whose overexpression increases resistance to infection by *Serratia marcescens*
T07H8.4	*mec-1*	protein with multiple disulfide-linked EGF and Kunitz domains
W02D3.3	*mec-6*	homolog of human PARAOXONASE 1 gene
C09G4.5	*mes-6*	WD repeat-containing protein
K01A2.8	*mps-2*	single-pass transmembrane protein that associate with and regulate pore-forming ion channels
F09G8.9	*mps-4*	single-pass transmembrane protein that associate with and regulate pore-forming ion channels
F14F4.3	*mrp-5*	homolog of the human gene CFTR
F53A2.8	*mtm-9*	Myotubularin
T08G5.10	*mtl-2*	one of the two metallothioneins which functions in metal detoxification, homeostasis and stress adaptation
K12F2.1	*myo-3*	MHC A, the minor isoform of myosin heavy chain
R107.1	*nac-2*	high affinity sodium-coupled citrate transporter
F33D4.1	*nhr-8*	nuclear hormone receptor homolog
ZK1058.2	*pat-3*	beta-integrin subunit
F23H11.8	*pef-1*	ortholog of the rhodopsin-specific serine/threonine phosphatase RdgC in *D. melanogaster*
K08E7.9	*pgp-1*	transmembrane protein required for defense against the pathogenic *Pseudomonas aeruginosa* strain PA14
C29E4.3	*ran-2*	homolog of RanGAP (GTPase activating protein)
E03A3.2	*rcq-5*	an ATP-dependent DNA helicase that is a member of the RecQ family of DNA helicases
R05D3.4	*rfp-1*	putative ubiquitin-protein ligase
C29H12.1	*rrt-2*	arginyl-tRNA synthetase
K01A2.1	*sgcb-1*	ortholog of human beta sarcoglycan
C32D5.2	*sma-6*	serine/threonine protein kinase
F46B6.3	*smg-4*	one of the seven smg genes mediating nonsense mediated decay or mRNA surveillance, resulting in the rapid decay of the aberrant transcript
Y54E5B.1	*smp-1*	semaphorin
R09G11.1	*sup-10*	predicted gene, no known function
F38H4.7	*tag-30*	predicted gene, no known function
C02F4.2	*tax-6*	ortholog of calcineurin A
T07C4.6	*tbx-9*	TBX2 and related T-box transcription factor
ZC21.2	*trp-1*	predicted transmembrane protein
C28G1.1	*ubc-23*	predicted conjugating enzyme of the ubiquitin-conjugation system
F30H5.1	*unc-45*	muscle-specific protein essential for proper thick filament formation and sarcomere organization
F45E10.1	*unc-53*	required for anteroposterior guidance of migrating cells and axons
F55C7.7	*unc-73*	guanine nucleotide exchange factor required for vulval morphogenesis
F08B6.4	*unc-87*	required to maintain the structure of myofilaments in body wall muscle cells
C09D1.1	*unc-89*	required for fully normal locomotion, pharyngeal muscle contractions and body size

C46F11.1	*unc-93*	novel transmembrane protein that affects muscle contraction and egg laying
T17A3.1	*ver-1*	member of the VEGF receptor family
F18C5.2	*wrm-1*	beta-catenin-like protein functioning in noncanonical Wnt signaling
F28F9.1	*zag-1*	homeodomain protein of the ZFH class
F08D12.7		novel protein conserved amongst *Caenorhaditis* species
F38B6.4		predicted gene, no known function
F41E7.6		predicted gene, no known function
F54C9.7		predicted gene, no known function
F55C7.2		predicted gene, no known function
R12E2.7		predicted gene, no known function

Querying a publicly accessible database (WormBase, release WS180), 81 genes reported to be expressed in nematode intestinal cells were selected and tested individually with RNAi for their abilities to modulate nematode survival during *B. thailandensis* infection.

Supplemental Table S3. List of plasmid constructs used in this study, related to all Figures.

Plasmid	Genotype or description	Source or reference
pL4440	Dual T7 RNAi feeding vector; ampicillin^r	CGC[a]
pGBKT7	GAL4 DNA-BD vector *[GAL4(1-147)]; TRP1*, kanamycin^r	Clontech[b]
pGADT7	GAL4 AD vector *[GAL4(768-881)]*; *LEU2*, ampicillin^r	Clontech[b]
pGBKT7-Daf2	DAF-2 C-terminus *[DAF2(1445-1843)* PCR-amplified with daf2-F (TggAgCTTCggAgTTgTTCT) and daf2-R (TATAAggATCCATAATTTAAgAggCgggATTTTT), digested with *Eco*RI and *Bam*HI and ligated in pGBKT7 vector*]*; *TRP1*, kanamycin^r	This study
pGADT7-Lin2	LIN-2 PDZ *[LIN2(288-647)* PCR-amplified with lin2-F (TATACATATggCgAgCAgAAAACACATgAA) and lin2-R (ATATCCCgggTggTTCgTAgTCgAATTgAgC), digested with *Nde*I and *Xma*I and ligated in pGADT7 vector*]*; *LEU2*, ampicillin^r	This study
pGBKT7-53	Positive control plasmid encoding fusion of murine p53 protein *[P53(72-390)]* and GAL4 DNA-BD *[GAL4(1-147)]*; *TRP1*, kanamycin^r	Clontech[b]
pGBKT7-Lam	Negative control plasmid encoding fusion of human lamin C protein *[LAM(66-230)]* and GAL4 DNA-BD *[GAL4(1-147)]; TRP1*, kanamycin^r	Clontech[b]
pGADT7-T	Positive control plasmid encoding fusion of SV40 large T antigen *[T(87-708)]* and GAL4 AD *[GAL4(768-881)]; LEU2*, ampicillin^r	Clontech[b]

[a] Caenorhabditis Genetics Center, University of Minnesota, Minneapolis, U.S.A.

[b] Clontech Laboratories Incorporated, California, U.S.A.

REFERENCES

Chávez, V., Mohri-Shiomi, A., Maadani, A., Vega, L.A. and Garsin, D.A. (2007) Oxidative stress enzymes are required for DAF-16-mediated immunity due to generation of reactive oxygen species by *Caenorhabditis elegans*. *Genetics*. **176**: 1567-1577.

Espelt, M.V., Estevez, A.Y., Yin, X. and Strange, K. (2005) Oscillatory Ca2+ signaling in the isolated *Caenorhabditis elegans* intestine: role of the inositol-1,4,5-trisphosphate receptor and phospholipases C beta and gamma. *J. Gen. Physiol.* **126**: 379-392.

Ferguson, E.L. and Horvitz, H.R. (1985) Identification and characterization of 22 genes that affect the vulval cell lineages of the nematode *Caenorhabditis elegans*. *Genetics*. **110**: 17-72.

Kawli, T., Wu, C. and Tan, M. (2010) Systemic and cell intrinsic roles of Gqalpha signaling in the regulation of innate immunity, oxidative stress, and longevity in *Caenorhabditis elegans*. *Proc Natl Acad Sci U S A*. **107**: 13788-13793.

Qadota, H., Inoue, M., Hikita, T., Köppen, M., Hardin, J., Amano, M.*, et al* (2007) Establishment of a tissue-specific RNAi system in *C. elegans*. *Gene*. **400**: 166-173.

Sim, B., Chantratita, N., Ooi, W., Nandi, T., Tewhey, R., Wuthiekanun, V.*, et al* (2010) Genomic acquisition of a capsular polysaccharide virulence cluster by non-pathogenic *Burkholderia* isolates. *Genome Biol.* **11**: R89.
