# Supplementary figures and images for: Modulation of Caenorhabditis elegans infection sensitivity by the LIN-7 cell junction protein
Source: Cell Microbiol. 2012 Jun 21;14(10):1584–99. doi: 10.1111/j.1462-5822.2012.01824.x (PMC3470699; doi:10.1111/j.1462-5822.2012.01824.x)

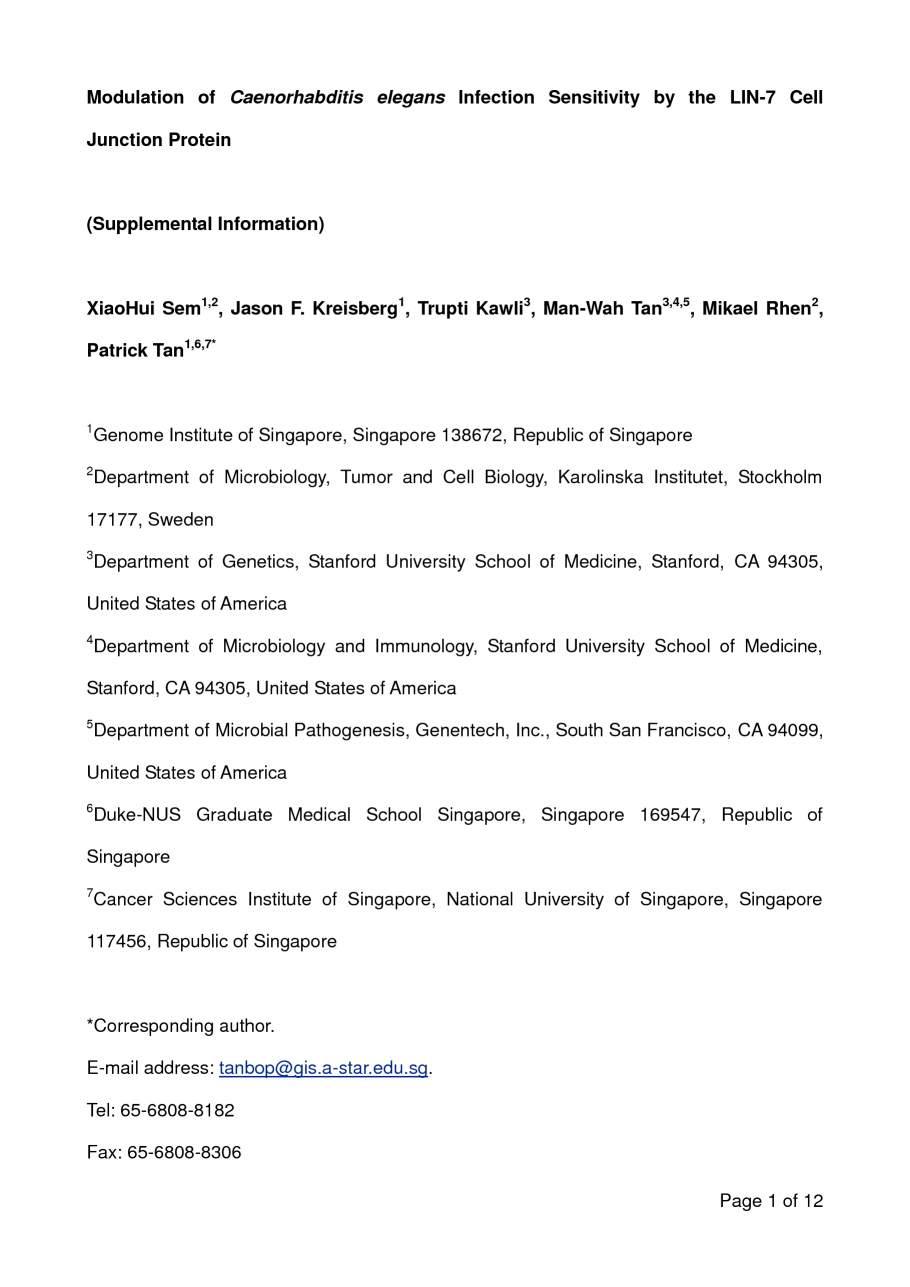

Supplement: Supplementary file 2 [file cmi0014-1584-SD2.png]
